# Horizontal transfer of exosomal microRNAs transduce apoptotic signals between pancreatic beta-cells

**DOI:** 10.1186/s12964-015-0097-7

**Published:** 2015-03-19

**Authors:** Claudiane Guay, Véronique Menoud, Sophie Rome, Romano Regazzi

**Affiliations:** Department of Fundamental Neurosciences, University of Lausanne, Rue du Bugnon 9, Lausanne, Switzerland; CarMeN Laboratory (INSERM U.1060/INRA 1397, INSA), University of Lyon, Faculty of Medicine Lyon-Sud, Ouillons, France

**Keywords:** Exosomes, MicroRNAs, Pancreatic beta-cells, Cell-to-cell communication, Diabetes

## Abstract

**Background:**

Diabetes mellitus is a common metabolic disorder characterized by dysfunction of insulin-secreting pancreatic beta-cells. MicroRNAs are important regulators of beta-cell activities. These non-coding RNAs have recently been discovered to exert their effects not only inside the cell producing them but, upon exosome-mediated transfer, also in other recipient cells. This novel communication mode remains unexplored in pancreatic beta-cells. In the present study, the microRNA content of exosomes released by beta-cells in physiological and physiopathological conditions was analyzed and the biological impact of their transfer to recipient cells investigated.

**Results:**

Exosomes were isolated from the culture media of MIN6B1 and INS-1 derived 832/13 beta-cell lines and from mice, rat or human islets. Global profiling revealed that the microRNAs released in MIN6B1 exosomes do not simply reflect the content of the cells of origin. Indeed, while a subset of microRNAs was preferentially released in exosomes others were selectively retained in the cells. Moreover, exposure of MIN6B1 cells to inflammatory cytokines changed the release of several microRNAs. The dynamics of microRNA secretion and their potential transfer to recipient cells were next investigated. As a proof-of-concept, we demonstrate that if *cel-miR-238*, a *C. Elegans* microRNA not present in mammalian cells, is expressed in MIN6B1 cells a fraction of it is released in exosomes and is transferred to recipient beta-cells. Furthermore, incubation of untreated MIN6B1 or mice islet cells in the presence of microRNA-containing exosomes isolated from the culture media of cytokine-treated MIN6B1 cells triggers apoptosis of recipient cells. In contrast, exosomes originating from cells not exposed to cytokines have no impact on cell survival. Apoptosis induced by exosomes produced by cytokine-treated cells was prevented by down-regulation of the microRNA-mediating silencing protein Ago2 in recipient cells, suggesting that the effect is mediated by the non-coding RNAs.

**Conclusions:**

Taken together, our results suggest that beta-cells secrete microRNAs that can be transferred to neighboring beta-cells. Exposure of donor cells to pathophysiological conditions commonly associated with diabetes modifies the release of microRNAs and affects survival of recipient beta-cells. Our results support the concept that exosomal microRNAs transfer constitutes a novel cell-to-cell communication mechanism regulating the activity of pancreatic beta-cells.

**Electronic supplementary material:**

The online version of this article (doi:10.1186/s12964-015-0097-7) contains supplementary material, which is available to authorized users.

## Background

Pancreatic beta-cells, located within the islets of Langerhans, play a central role in the regulation of blood glucose homeostasis by secreting insulin in response to the organism demand. Insufficient insulin supply, resulting from beta-cell dysfunction and/or death, leads to chronic hyperglycemia and favor the development of diabetes mellitus, the most common metabolic disorder worldwide. Proper functioning of insulin-secreting cells is governed by a complex array of signals of metabolic, hormonal and neuronal origin that ensure a tight control of insulin release. MicroRNAs (miRNAs), a class of non-coding RNAs, have been identified as important determinants of the functional integrity of pancreatic beta-cells. These small RNA molecules regulate not only the differentiation of beta-cells but also various aspects of the activity of mature beta-cells including proliferation, survival, insulin biosynthesis and secretion [1-4]. miRNAs act by binding to the 3’UTR of target mRNAs of specific genes leading to translational repression and/or to a decrease in messenger stability [5]. Beside their activity accomplished inside the cell producing them, these small RNA molecules can also be released in the extracellular environment packaged within microvesicles or in association with proteins or high-density lipoproteins (HDL) [6-8]. The role of circulating miRNAs remains to be ascertained but, since miRNAs transported by HDL or exosomes can be transferred in active form to other cells, they have been suggested to represent a novel cell-to-cell communication mode [8,9].

Exosomes are microvesicles of 50–150 nm of diameter that originate from the late endosomal pathway and are secreted in the extracellular space upon fusion of multivesicular bodies with the plasma membrane [10]. The biogenesis and release of exosomes have been shown to involve different mechanisms including the ceramide pathway, the ESCRT system and the exocytotic machinery [11-13]. Exosomes accumulate in the culture media of several cell types, and are present in different body fluids such as blood, urine or saliva [14]. These extracellular vesicles carry proteins and nucleic acids, including miRNAs that can be transferred to recipient cells [9,15]. Little is known about the sorting of proteins and RNAs into exosomes, but horizontal transfer of the exosomal cargo to neighboring cells has been suggested to allow rapid phenotype adjustment in response to changes in physiological or physiopathological conditions [16]. RISC (RNA Induced Silencing Complex) components, which are essential for miRNA action, are also present in exosomes [7]. Several cell types have been shown to release miRNAs that can be transferred in active form to recipient cells, where they can exert their regulatory activity on target genes [9,17,18]. Exosomal miRNAs were reported to be involved in a broad range of biological processes including the immune reaction and in the regulation of the cardiovascular system [19,20].

Pancreatic islets and different beta-cell lines have been reported to release exosome-like microvesicles [21-25]. The function of beta-cell exosomes is just beginning to unfold, but there is already evidence indicating that these extracellular vesicles may participate in the cross-talk with endothelial cells or lymphocytes [21,24]. The possible contribution of exosomal miRNAs in the autocrine signaling between pancreatic beta-cells has, however, so far not been explored.

In the present study, we investigated if the release of exosomes from pancreatic beta-cells can affect the surrounding cells and can constitute a cell-to-cell communication mode permitting a concerted adaptation of beta-cells to environmental cues. Our data indicate that certain miRNAs are preferentially secreted in exosomes while others are specifically retained inside beta-cells. Moreover, we found that the miRNA content of exosomes is modified upon exposure of the beta-cells to inflammatory mediators. Interestingly, incubation with exosomes originating from cells treated with cytokines induced apoptosis of naïve beta-cells, an effect that was prevented by inhibition of Ago2, a component of the RISC complex that is essential for miRNA action, in recipient beta-cells. Taken together, these results suggest that exosome-mediated transfer of miRNAs constitute a novel communication mode between pancreatic beta-cells.

## Results

### Isolation and characterization of exosomes released by pancreatic beta-cells

**Figure 1:**
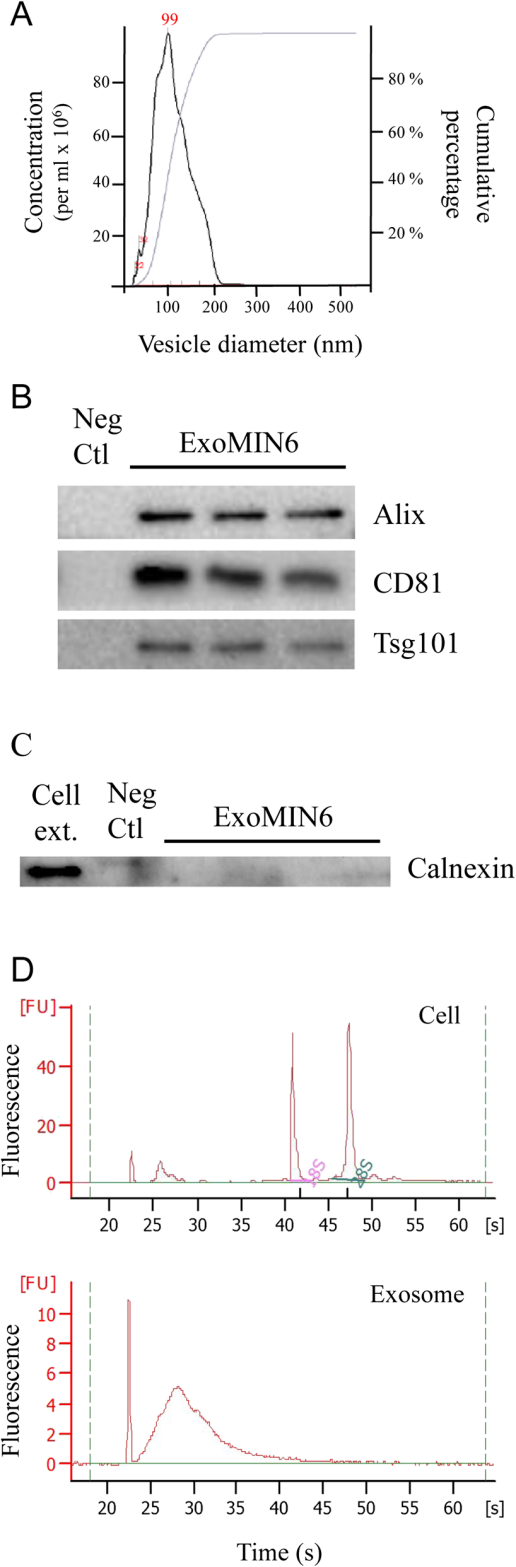
**Characterization of exosomes released by MIN6B1 cells. A)** The sizes of microvesicles isolated by ultra-centrifugation from the culture media of MIN6B1 cells was measured using the Nanosight technology. **B, C)** Three different preparations of microvesicles from MIN6B1 cells were analyzed by immunoblotting for **B)** the presence of the exosomal protein markers Alix, CD81 and Tsg101, and **C)** for the absence of the cellular protein Calnexin. Cell extract was used as positive control for the detection of Calnexin. **D)** RNA profiles of MIN6B1 cells (upper panel) and of microvesicles (lower panel) were determined by Bioanalyzer.

Microvesicles released by the pancreatic beta-cell line MIN6B1 cultured in DMEM medium complemented with exosome-depleted FCS (see Methods) were isolated by ultra-centrifugation and characterized for their size and content. Using Nanosight technology, the purified microvesicles were found to display a diameter comprised between 50 and 200 nm (Figure 1A). Moreover, the microvesicles were enriched for the exosome markers Tsg101, Alix and CD81 (Figure 1B). In contrast, the endoplasmic reticulum protein Calnexin was undetectable in exosomes, suggesting that no or very little amount of cell debris was present in our preparations (Figure 1C). These results indicate that microvesicles isolated from the culture media of MIN6B1 cells consist mainly of exosomes and will be referred as such hereafter.

**Figure 2:**
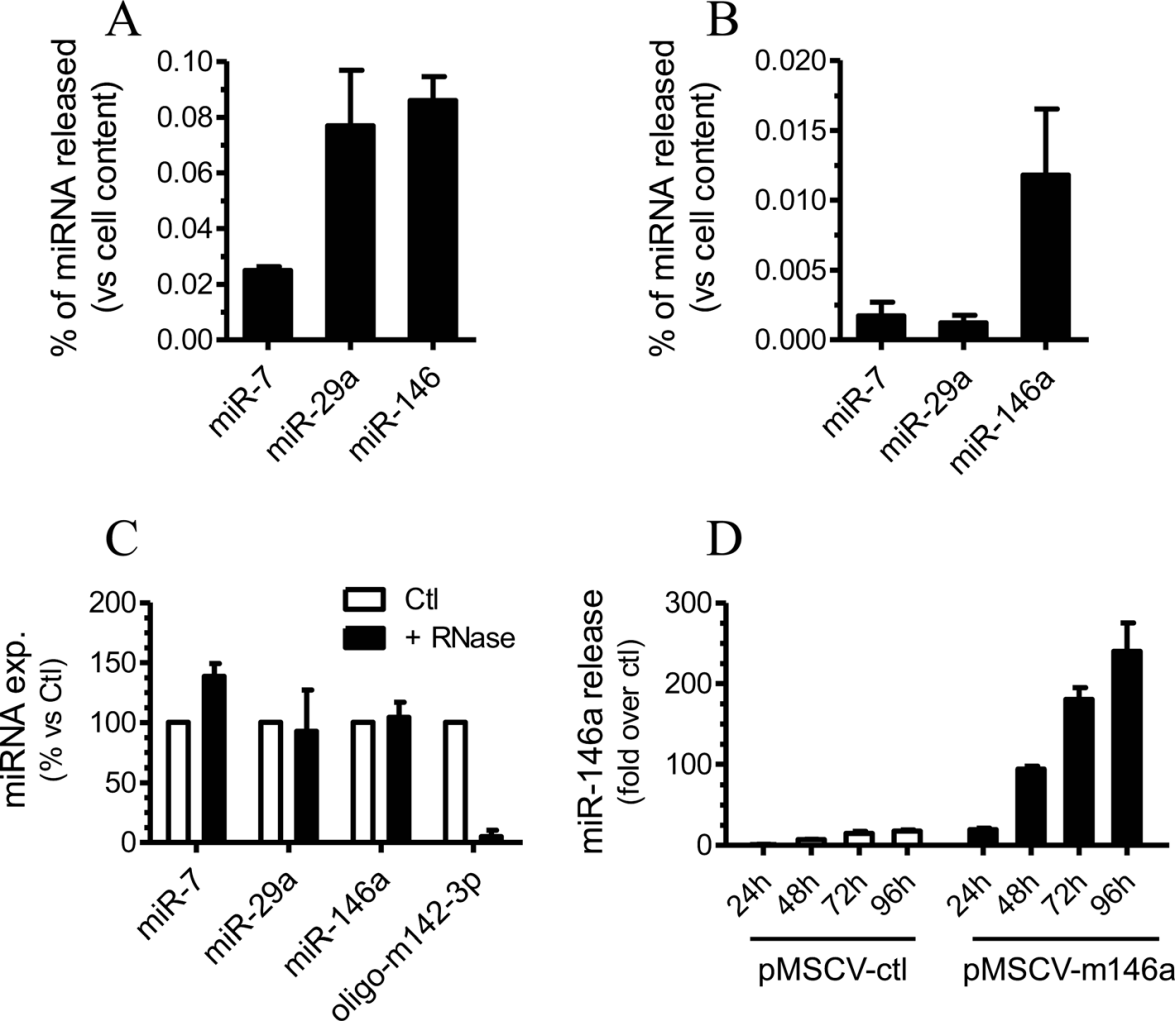
**miRNAs are released in exosomes of beta-cells.** RNA was isolated from exosomes purified from the culture media of MIN6B1 cells **(A)** and human islets **(B)**. The amount of miR-7, miR-29a and miR-146a recovered in exosomes was measured by qPCR and is expressed as percentage of the corresponding miRNA present inside MIN6B1 cells **(A)** or human islets **(B). C)** Exosomes isolated from MIN6B1 culture media were spiked with an oligonucleotide corresponding to the sequence of miR-142-3p (oligo-m142-3p) and incubated with (+ RNase) or without (Ctl) RNAse A and T1. **D)** MIN6B1 cells were transfected with a control pMSCV plasmid (pMSCV-ctl) or with a plasmid coding for miR-146a (pMSCV-m146a). Media were collected 24 h, 48 h, 72 h and 96 h after transfection. Exosomes were isolated and analyzed for their miRNA content.

The total RNA content of exosomes released by MIN6B1 cells (exoMIN6) was next analyzed using a Bioanalyzer. As shown in Figure 1D, exoMIN6 carried mainly RNAs of small sizes (lower panel) whereas, as expected, MIN6B1 cell extracts contained larger RNA molecules, including 18S and 28S RNAs (upper panel). The presence of specific miRNAs in exosomes was investigated by qPCR analysis. As shown in Figure 2, miRNAs known to be expressed at high level in beta-cells [26-28], such as miR-7, miR-29a and miR-146a, were released in exosomes by MIN6B1 cells and human islets. Over a period of 72 h, MIN6B1 cells were estimated to release in exosomes between 0.02–0.09% of the cellular content of these miRNAs and between 0.002 and 0.012% of these miRNAs was recovered in exosomes after incubation of human islets for 24 h. These three miRNAs were also present in exosomes isolated from the culture media of INS-1 832/13 cells and of mice and rats islets. As expected, miR-7, miR-29a and miR-146a released by MIN6B1 cells were protected from RNase treatment confirming that they resided inside the exosomes (Figure 2C). In contrast, the same RNase treatment degraded an oligonucleotide mimicking the sequence of miR-142-3p directly spiked in the exosome preparations. We next investigated the kinetics of the release of the miRNAs from beta-cells (Figure 2D). MiR-146a was readily detectable in the culture media of MIN6B1 cells after 24 h of culture, and its level steadily increase upon longer incubation periods. Overexpression of miR-146a (Additional file 1: Figure S1) resulted in a time-dependent elevation of the level of this miRNA recovered in the exosome preparations (Figure 2D). Taken together, these results indicate that pancreatic beta-cells constantly release exosomes containing miRNAs.

### Effect of physiopathological conditions on the pool of miRNAs released by pancreatic beta-cells

**Figure 3:**
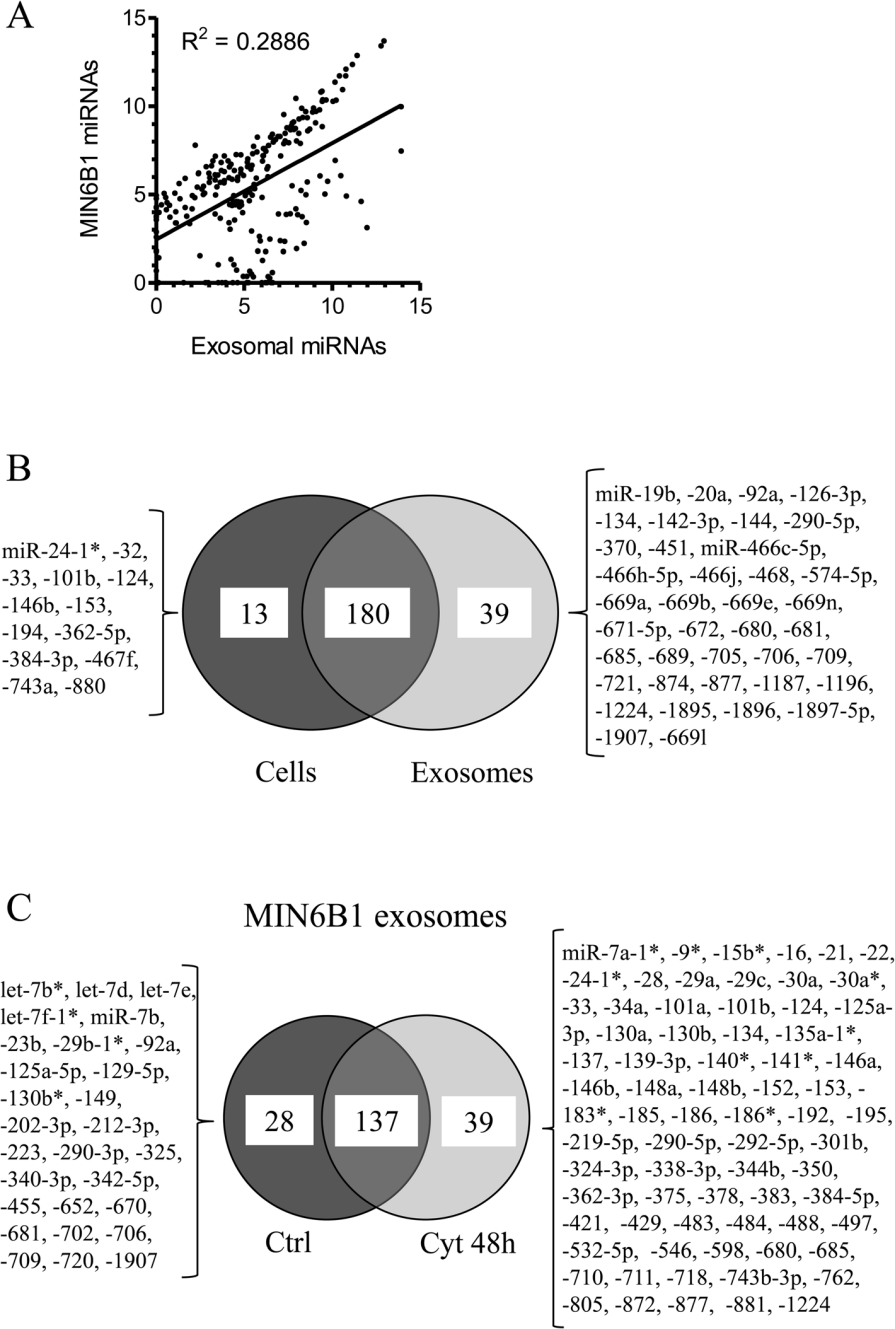
**Profiling of miRNAs released in exosomes by MIN6B1 cells in the presence or absence cytokines.** miRNA content of exosomes released by MIN6B1 cells treated or not with pro-inflammatory cytokines (IFNγ, TNF-α and IL-1β) for 48 h were analyzed by microarray and were compared to the cellular content (complete microarray results are presented in Additional file 2: Table S1). **A)** Comparison of the amount of each miRNA released in exosomes and retained inside MIN6B1 cells under control conditions. **B)** Venn diagram showing the miRNAs most enriched in exosomes or in cellular extracts (fold change > 15; corrected *p* value < 0.05). Of the 232 miRNAs detected, 39 were preferentially found in exosomes, whereas 13 miRNAs were more abundant inside the cells. **C)** Venn diagram presenting exosomal miRNAs displaying significant changes in response to cytokine treatment (fold change > 1.5; corrected *p* value < 0.05).

To obtain a global view of the miRNAs released by beta-cells, we compared by microarray the level of these small RNAs present inside MIN6B1 cells and released in exosomes (see Methods for the microarray analysis details). Of the 650 miRNAs tested, 232 miRNAs were detectable in the cells and/or in the exosomes (Additional file 2: Table S1). Interestingly, we found that the level of the miRNAs present in the exosomes is not simply the reflection of their abundance inside the cells (Figure 3A). Indeed, although the relative abundance of several miRNAs was comparable in cell and exosome extracts, some miRNAs were preferentially released in exosomes (Figure 3B). For example, miR-451 and miR-142-3p were much more abundant in exosomes than in MIN6B1 cell extracts, whereas the levels of miR-32 and miR-194 were clearly higher inside the cells. These findings were confirmed by qPCR (Additional file 3: Figure S2A).

We also examined if exposure to physiopathological conditions known to favor the development of diabetes mellitus affect the pool of miRNAs released by beta-cells (Additional file 2: Table S1). Interestingly, treatment of MIN6B1 cells with a mix of pro-inflammatory cytokines, including IL-1β, TNFα and IFNγ, changed the level of 67 of the 204 miRNAs detected in exosomes (p < 0.05; Fold change > 1.5). Indeed, the level of 28 miRNAs diminished in exosomes of MIN6B1 cells treated with cytokines whereas 39 of them were present at higher levels (Figure 3C). For example, miR-546 and miR-710 were increased in response to cytokines whereas let-7e and miR-212-3p were more abundant in exosomes of untreated MIN6B1 cells (see Additional file 3: Figure S2B for confirmation of these results by qPCR). Interestingly, among the miRNAs found to be upregulated in exosomes in response to cytokines, several of them including miR-146a, miR-146b, miR-195, miR-290a-3p, miR-362-3p and miR-497 are known to be involved in cell death [29-34].

### Exosomes released during cytokine exposure affect survival of receiving beta-cells

**Figure 4:**
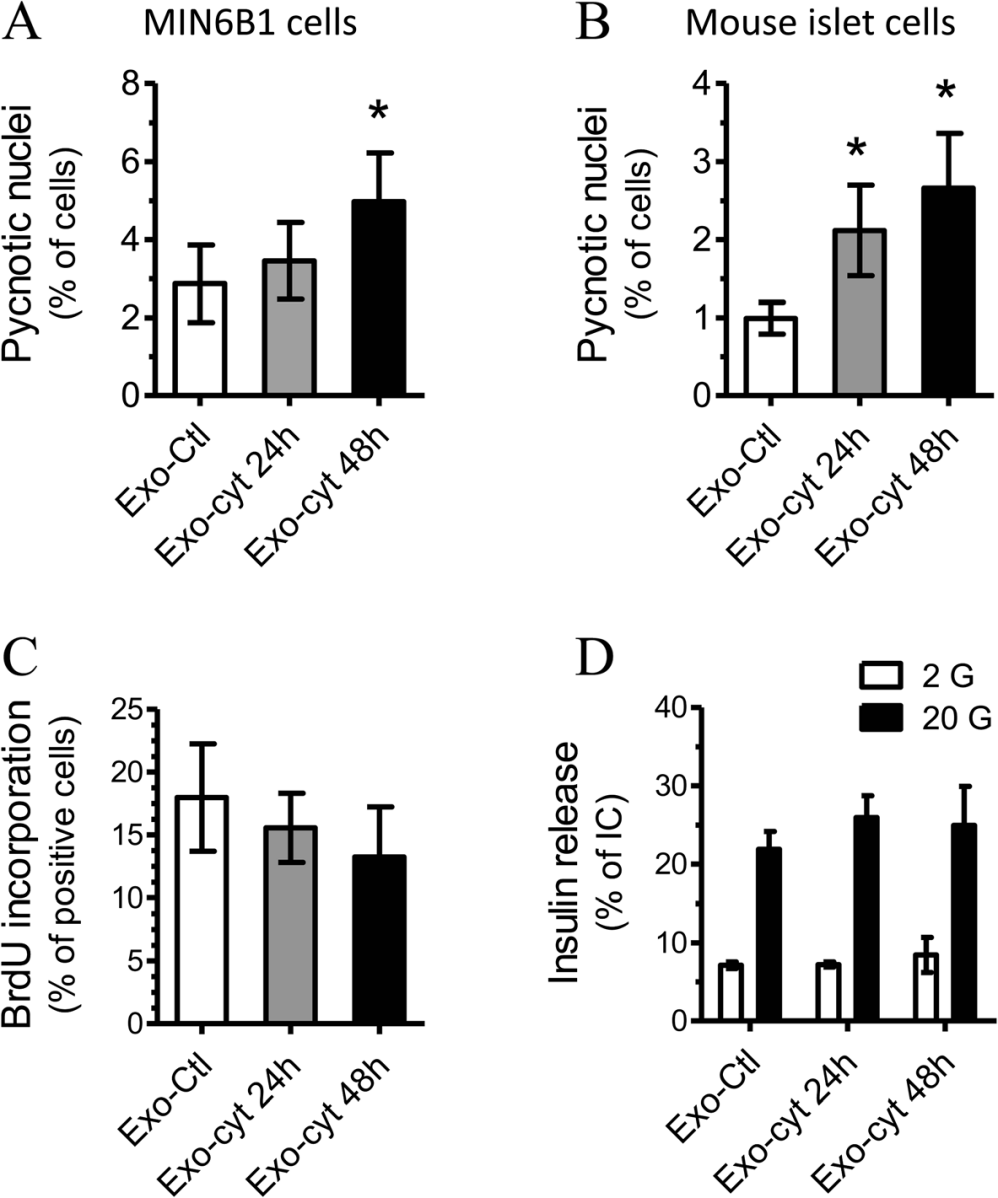
**Exosomes from cytokine-treated cells induce apoptosis of recipient naïve beta-cells.** Exosomes were isolated from the media of MIN6B1 cells cultured for a total of 48 h and treated with a combination of pro-inflammatory cytokines (IFNγ, TNF-α and IL-1β) for 0 h (Exo-Ctl), 24 h (Exo-cyt 24 h) or 48 h (Exo-cyt 48 h). Recipient naïve MIN6B1 cells **(A, C, D)** or dispersed mouse islet cells **(B)** were incubated with the different exosome populations for 72 h and then functionally characterized. **A, B)** Cell death was assessed by scoring the cells displaying pycnotic nuclei upon Hoechst staining. **C)** Beta-cell proliferation was assessed by BrdU incorporation. **D)** Insulin secretion at 2 or 20 mM glucose was measured by ELISA and was expressed as percentage of insulin content (IC). The results are means ± SD of at least four independent experiments. *Significantly different from control condition (Exo-Ctl), p ≤ 0.05 by ANOVA analysis, Dunnett’s post-hoc test.

Exosomes have recently been proposed to play important roles in cell-to-cell communication [16]. Therefore, we explored if the transfer of the exosome content from a beta-cell to its neighbors can transmit a signal of biological relevance. To test this hypothesis, we purified exosomes from the culture media of MIN6B1 cells treated or not with cytokines. Protein content of the different exosome preparations were similar (Exo-Ctl: 22.7 +/− 6.3 μg, Exo-24 h: 23.4 +/− 3.0 μg, Exo-48 h: 27.7 +/− 4.4 μg) suggesting that cytokine treatment did not affect the amount of exosomes released by MIN6B1 cells. Interestingly, incubation of naïve MIN6B1 or dispersed mouse islet cells in the presence of exosomes originating from donor cells exposed to cytokines led to a significant increase in apoptosis (Figure 4A, B). In contrast, the exosomes purified from the medium of untreated MIN6B1 cells did not affect the survival of recipient cells (Additional file 4: Figure S3A). The apoptotic effect is not mediated by cytokines or other soluble factors carried over during the isolation procedure since incubation of recipient MIN6B1 cells with the supernatants recovered after ultracentrifugation of the exosome preparation (i.e. the medium in which the exosomes are suspended) did not affect cell survival (Additional file 4: Figure S3B). A trend to a reduction in cell proliferation was also observed (Figure 4C). However, incubation of MIN6B1 cells in the presence of exosomes did not affect insulin release in response to glucose (Figure 4D) nor the total cellular insulin content (data not shown).

### Exosome-induced apoptosis in recipient beta-cells is mediated by miRNA transfer

**Figure 5:**
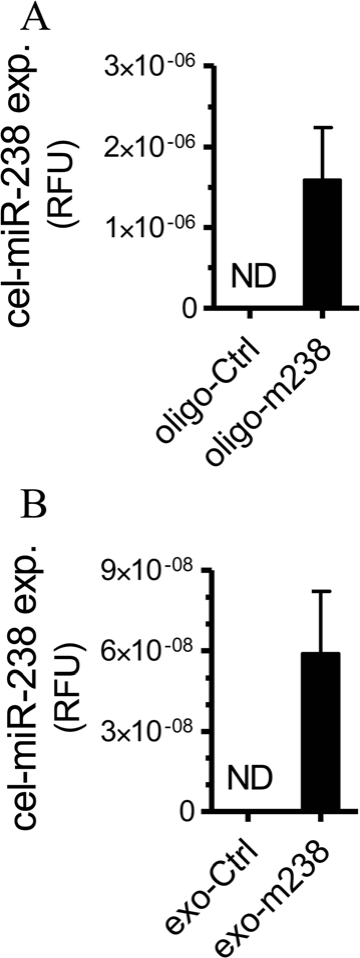
**Horizontal transfer of** ***cel-miR-238*** **between MIN6B1 cells. A)** Donor MIN6B1 cells were transfected with a control oligonucleotide (oligo-Ctrl) or with an oligonucleotide corresponding to the sequence of *cel-miR-238* (oligo-m238). **B)** Recipient MIN6B1 cells were incubated in presence of exosomes from donor cells transfected with control (Exo-Ctrl) or with miR-238 (Exo-m238) oligonucleotides. The level of *cel-miR-238* was measured by qRT-PCR in both donor and recipient cells. (ND = below detection limit of qPCR).

We next investigated whether the mechanism leading to beta-cell death in recipient MIN6B1 cells involves an exosome-mediated transfer of miRNAs. As a proof of concept, MIN6B1 donor cells were transfected with an oligonucleotide mimicking the sequence of miR-238 from *Caenorhabditis elegans* (*cel-miR-238*), a miRNA that is normally not expressed in MIN6B1 cells (Figure 5A). Exosomes isolated from the culture media of cells expressing *cel-miR-238* (exo-m238) or from control cells (exo-Ctrl) were added to the culture media of recipient naïve MIN6B1 cells. As shown in Figure 5B, recipient MIN6B1 cells incubated in the presence of exo-m238 were found to contain *cel-miR-238*, demonstrating the possibility of a horizontal transfer of miRNAs between pancreatic beta-cells. As expected, *cel-miR-238* was not detected (ND) in MIN6B1 cells transfected with a control oligonucleotide or incubated with exo-Ctrl (Figure 5 A-B).

**Figure 6:**
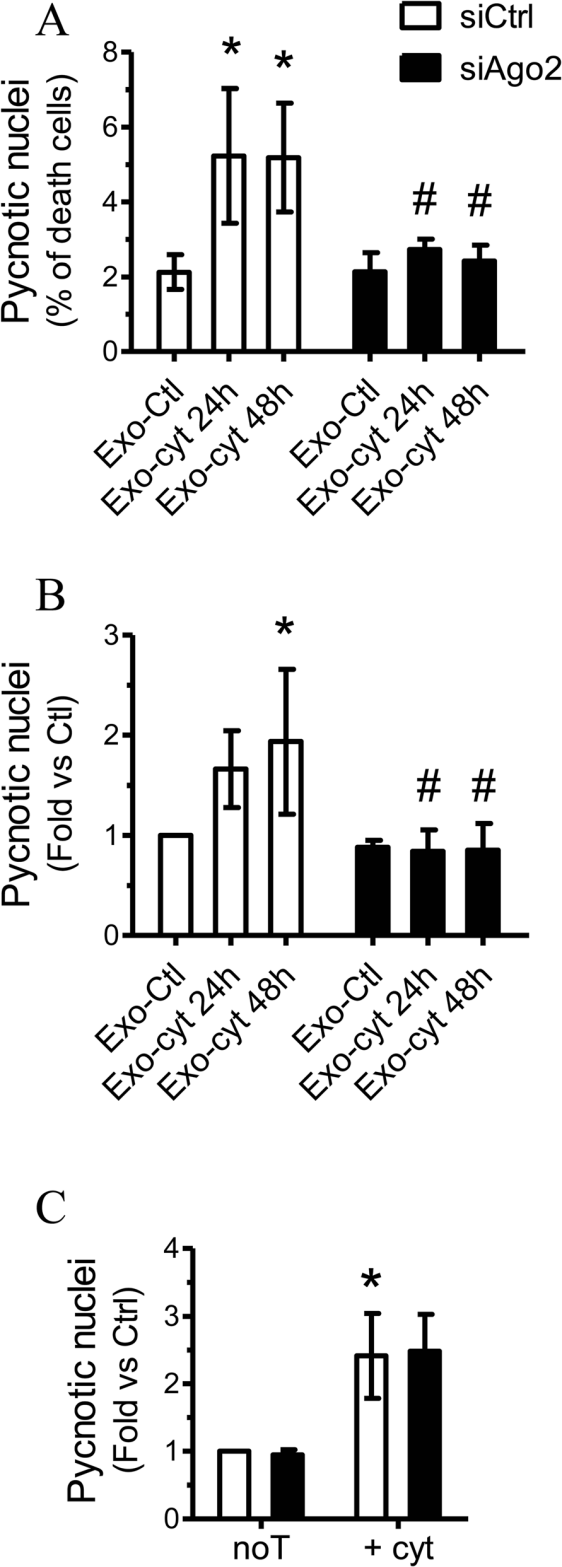
**Ago2 inactivation in recipient beta-cells prevents apoptosis induced by exosomes of cytokine-treated cells.** Exosomes were isolated from the media of MIN6B1 cells treated for 0 h (Exo-Ctl), 24 h (Exo-cyt 24 h) or 48 h (Exo-cyt 48 h) with cytokines. Recipient naïve MIN6B1 **(A)** or dispersed mouse islet cells **(B)** were transfected with siCtrl or siAgo2 and were then incubated for 72 h with the different exosome preparations. **C)** MIN6B1 cells were transfected with siCtrl or siAgo2 and incubated for 72 h. Cytokines (+cyt) were added to the culture media for the last 24 h of incubation. Apoptosis was assessed at the end of the incubation time by scoring the cells displaying pycnotic nuclei upon Hoechst staining. The results are means ± SD of at least four independent experiments. *Significantly different from respective control condition (siCtrl-Exo-Ctl or siCtrl-NoT), # Significantly different from the respective control condition (siCtrl-Exo-cyt24h or siCtrl-Exo-cyt48h). p ≤ 0.05 by ANOVA analysis, Tukey’s post-hoc test.

If the observed increase in apoptosis is mediated by a transfer of miRNAs, then inactivation of these small non-coding RNAs in recipient cells should prevent exosome-induced cell death. To test this hypothesis, recipient MIN6B1 (Figure 6A) or dispersed mouse islet cells (Figure 6B) were transfected with a siRNA directed against Argonaute 2 (siAgo2) (Additional file 5: Figure S4), a component of the RISC complex that is essential for miRNA action [5]. Under control conditions, transfection of MIN6B1 (Figure 6A) or dispersed islet cells (Figure 6B) with siAgo2 did not affect cell survival. However, transfection of recipient beta-cells with siAgo2 prevented apoptosis induced by exosomes originating from MIN6B1 donor cells treated with cytokines (Exo-cyt). Similar results were obtain when exosomes were isolated from cytokine-treated MIN6B1 cells cultured in DMEM media containing exosome-free FCS (Additional file 6: Figure S5). This protective effect was specific to cell death induced by Exo-Cyt since siAgo2 failed to prevent apoptosis in response to cytokines (Figure 6C). Taken together, these results suggest that horizontal transfer of miRNAs via exosomes produced by beta-cells exposed to pro-inflammatory cytokines can affect survival of surrounding cells.

## Discussion

Coordinated activity of pancreatic beta-cells located within the islets of Langerhans is essential to insure a tight control of blood glucose levels and to prevent the deleterious effects of hypo- or hyperglycemia. This coordination is achieved both by direct cell-to-cell contact, mediated by cell adhesion molecules or gap junctions, and through the release of a variety of signaling molecules with paracrine or autocrine functions [35,36]. In the present study, we investigated the biological relevance of a novel signaling mechanism involving the horizontal transfer of miRNAs between pancreatic beta-cells. We found that miRNAs carried by exosomes do not simply reflect the cellular content in physiological or physiopathological conditions. Our results rather suggest that particular miRNAs are preferentially released in exosomes while others are selectively retained by the cells. In agreement with our observation, some miRNAs including miR-126, miR-139, miR-223 and miR-483 were found to be enriched by more than 10 fold compared to the cellular content in exosomes originating from human islets [21]. Selective accumulation of specific miRNAs in exosomes released by other cell types was also reported [7,9,15,37,38]. The mechanisms governing the loading of miRNAs in exosomes remain unknown, but recent publications tried to unravel them. Villarroya-Beltri *et al.* [39] reported enrichment in exosomes of T lymphocytes of miRNAs containing the GGAG sequence that binds to the ribonucleoprotein hnRNPA2B1. They found that sumoylation regulates the binding of hnRNPA2B1 to miRNAs and favors the trafficking of the non-coding RNAs toward the exosomes. Squadrito *et al.* noted an inverse correlation in macrophages between the level of the miRNAs sorted in exosomes and the abundance of their target transcripts in the cells [40]. This finding suggests that changes in the level of the mRNAs targeted by a given miRNA can influence its re-localization from cytoplasm/P-bodies to multivesicular bodies and hence its release in the extracellular space. More studies are needed to confirm these findings in different cell types and to investigate whether the motifs and/or pathways directing the intracellular trafficking of miRNAs are universally conserved or are specific for each cell type.

We observed that incubation of MIN6B1 cells in the presence of pro-inflammatory cytokines, a condition typically encountered in pre-diabetic and diabetic states, modifies the profile of the miRNAs carried by exosomes. Incubation of naïve beta-cells with exosomes from donor cells treated with cytokines does not affect the secretory functions of the recipient cells but results in an increase in apoptosis. During the preparation of this manuscript, Zhu and colleagues published a study demonstrating that incubation of INS1 cells, another beta-cell line, in the presence of low doses of cytokines release exosomes capable of protecting the recipient cells from cytokine-induced cell death [25]. These authors did not test the functional impact of exosomes produced from INS1 cells treated with higher doses of cytokines and they have not investigated the potential role of miRNAs in the protective activity of these extracellular vesicles. The effect of pro-inflammatory cytokines on beta-cells is known to be concentration dependent. Low doses of cytokines favor the activity and survival of the cells whereas at higher concentrations they promote cell death [41,42]. It is possible that this dual effect of cytokines would be reflected in the release of different types of exosomes exerting either protective or deleterious impacts on the survival of the receiving cells according to the concentration of cytokines experienced by the donor cells. Should this hypothesis be confirmed, exosomes would represent a novel cell-to-cell communication mode permitting a concerted response to inflammatory mediators. The regulation of proliferation by paracrine signals has been less studied, but there is indication suggesting that extracellular vesicles may be implicated also in this phenomenon. Indeed, in a model of HNF1a deficiency, Bonner *et al.* observed that apoptotic INS1 cells release microparticles that stimulate proliferation of naïve recipient INS1 cells [43]. This proliferative signal was prevented by filtration of the conditioned media, suggesting that it is mediated by microparticles of relatively large sizes (>0.22 μm) rather than by exosomes or soluble factors. It is not yet known whether or not this proliferative effect is linked to the transfer of miRNAs or of other regulatory molecules transported by the microparticles.

The precise cascade of events elicited by exosomes and culminating in beta-cell apoptosis remains to be elucidated. Our data suggest that miRNAs may play a role in the effect of exosomes on beta-cell survival. Indeed, we demonstrated that the incubation of donor cells with cytokines alters the pool of released miRNAs and that exosomal miRNAs can be delivered to recipient beta-cells. Moreover, silencing of Ago2, a component of the RISC complex that is essential for miRNA action, in recipient cells prevents the apoptotic effect of exosomes originating from cells exposed to cytokines. Ago2 is known to be present also in exosomes [7]. However, our data indicate that the transferred miRNAs rely mainly on the RISC complex of the recipient cells to induce cell death. Taken together, our observations suggest that the apoptotic signal may by transduced by the miRNAs carried within the exosomes. However, we cannot exclude that other components of the exosomes elicit secondary changes in miRNA expression in recipient beta-cells contributing to the deleterious impact on cell survival.

## Conclusions

This study highlighted a novel category of signals generated by pancreatic beta-cells exposed to pro-inflammatory mediators. The exosomes released by beta-cells under these conditions will not only permit to achieve a coordinated response to inflammation of neighboring beta-cells but may also alert the immune system and favor the activation of an autoimmune reaction culminating in the development of type 1 diabetes [24]. Thus, the identification of the molecular mechanisms underlying this novel cell-to-cell communication mode and, in particular, a precise definition of the role played by miRNAs in this process will possibly help the design of new strategies to prevent or treat diabetes.

## Methods

### Pancreatic beta-cell lines

The murine insulin-secreting cell line MIN6B1 [44] (passage 15–32) was cultured in DMEM- GlutaMAX^TM^ medium (Gibco) containing 25 mM of glucose and 4 mM of L-glutamine and supplemented with 15% FCS, 50 U/ml penicillin, 50 μg/ml streptomycin, and 70 μmol/L ß-mercaptoethanol (referred to as full DMEM media in the text). For exosome characterization and analysis by microarray and qPCR, FCS was pre-centrifuged at 100’000 x *g* for 2 h to remove bovine exosomes. Rat insulinoma INS-1 derived 832/13 cells [45] (passages 50–62) were cultured in RPMI 1640- GlutaMAX^TM^ medium (Gibco) containing 11.1 mM glucose and 2 mM L-glutamine and supplemented with 10% fetal calf serum (Amimed), 10 mM HEPES, 1 mM sodium pyruvate and 50 μM β-mercaptoethanol (referred to as complete RPMI media in the text). For exosome isolation, MIN6B1 cells were seeded at 4 x 10^6^ cells and cultured for 48 h in full DMEM media. To evaluate the effect of pro-inflammatory cytokines, the medium included 30 ng/ml IFNγ (R&D systems), 10 ng/ml TNFα (R&D systems) and 1 ng/ml IL-1β (Sigma-Aldrich) from the beginning of the culture period or during the last 24 h of incubation. Both cell lines were cultured at 37°C in a humidified atmosphere (5% CO_2_, 95% air).

### Human islets

Human islets were obtained from the Cell Isolation and Transplantation Center of the University of Geneva, though the ECIT “Islets for Research” distribution program sponsored by the Juvenile Diabetes Research Foundation. The use of human islets for research was approved by the local institutional ethical committee. For exosome isolation, human islets were cultured in CMRL medium (Gibco) supplemented with 10% FCS, 100 U/ml penicillin, 100 μg/ml streptomycin, 2 mM glutamine, and 250 μM HEPES for up to 48 h.

### Rodent islets

Male Wistar rats (250 g) and male C57Bl/6 N mice (11–13 weeks-old) were obtained from Charles River Laboratories (L’arbresle, France). All animal procedures were performed in accordance with the NIH guidelines and protocols were approved by the Swiss Research Councils and Veterinary Offices. Rodent islets were isolated by collagenase digestion of the pancreas [46] followed by Histopaque density gradient and hand-picking to separate pancreatic islets from digested exocrine tissue. Isolated islets were incubated overnight in complete RPMI 1640- GlutaMAX^TM^ medium without β-mercaptoethanol and supplemented with 100 μg/mL streptomycin and 100 IU/mL penicillin, at 37°C in a humidified atmosphere (5% CO_2_, 95% air). After overnight recovery, media were collected for exosome isolation and islets were dissociated by incubation for 3 min at 37°C in Ca^2+^/Mg^2+^ free PBS containing 3 mM EGTA and 0.002% trypsin, with gentle shaking by pipetting.

### Exosome isolation

Exosomes were isolated by ultra-centrifugation as described previously [47]. Briefly, culture media of MIN6B1 or INS 832/13 cells or from mouse, rat or human islets were collected and centrifuged first at 300 *g* for 5 min to pellet the intact cells and then at 2’000 *g* for 10 min to discard the dead cells. Supernatants were then centrifuged at 10’000 *g* for 30 min to remove cell debris. Exosomes were isolated from the final supernatant by ultra-centrifugation at 100’000 *g* for 2 h. The pellet containing the exosomes was washed with PBS and re-centrifuged at 100’000 *g* for 2 h. Exosomes were collected in a minimal volume of PBS and stored at -80°C. Exosomes isolated from MIN6B1 treated with cytokines were diluted in PBS to a final volume corresponding to 1:100 of the original culture volume. Protein concentration was determined by Bradford assay (BioRad). MIN6B1 recipient cells were incubated for 72 h with exosome preparations at a final concentration of 50 μg/ml [21]. To evaluate the impact of small amounts of soluble molecules potentially carried over during the isolation procedure, recipient cells were incubated with the same volume of supernatant but depleted from exosomes. Survival and functional assays were then performed as described below.

### Nanosight analysis

Exosome size distribution was determined by Nanoparticle Tracking Analysis using the NanoSight system (NanoSight, UK). This technique measures the Brownian motion of particles for which the speed of movement, or diffusion coefficient, is related to particle size through the Stokes-Einstein. Filtered PBS was used as solvent to dilute exosomes.

### Western blotting

Proteins from exosome samples or cell lysates were migrated on 10% SDS-PAGE gels. Following electrophoresis, the proteins were transferred to PVDF membranes that were blocked at room temperature in Tris-buffered saline/0.3% Tween-20 containing 4% of BSA. Membranes were then incubated overnight at 4°C with gentle shaking with antibodies against CD81 (sc-166028), Alix (sc-49268) or TSG101 (sc-6037), purchased from Santa Cruz Biotechnology, or against Calnexin (S0998) obtained from Epitomics. All antibodies were diluted 1/1000 in 1% BSA. The signal was detected using a horseradish peroxidase-conjugated secondary antibody (BioRad) and was revealed with the enhanced chemiluminescence system from Pierce.

### RNA extraction from beta-cells and exosomes

Total RNA from cell or exosome preparations was extracted using the miRNeasy kit (Qiagen) and quantified with a NanoDrop1000 spectrophotometer (Witec AG). The size of the RNAs isolated from MIN6B1 cells or present in exosomes was analyzed using a Bioanalyzer (Agilent Technology).

### Quantification of mature miRNA levels

Expression of mature miRNAs was quantified with the miRCURY LNA^TM^ Universal RT microRNA PCR kit (Exiqon). Briefly, 200 ng of exosomal or cellular RNAs were used for reverse transcription (RT) in a final volume of 20 μl. Each RT reaction was diluted 10 times in RNase-free water and 8 μl of the cDNA template was combined to the ExiLENT SYBRgreen master mix. qPCR reactions were carried out in triplicates using the CFX Real-Time PCR Detection System (BioRad). miRNA expression in exosomes was normalized to the amount of RNA and expressed as Relative Fluorescence Units (RFU) or compared to control condition whenever possible. The UniSp6 RNA spike-in control (Exiqon) was used as additional internal reference. For this purpose, 0.15 fmol of UniSp6 (corresponding to about 10^8^ copies) was added to the RT reaction and was measured by qPCR using specific primers. miRNA expression in beta-cells was normalized to U6 content.

### RNase treatment

After isolation, exosomes were collected in 150 μl PBS and 20 ρmol of an oligonucleotide duplex containing the mature miR-142-3p sequence (Eurogentec) were spiked in each sample. Samples were then divided in two aliquots. RNase A (0.5 U) and RNase T1 (15 U) enzymes (Ambion) were added to the first set of tubes while the others were incubated without the enzymes. RNase digestion was performed for 30 min at 37°C. At the end of the incubation, RNA was extracted as described above.

### Cell transfection

MIN6B1 cells were transiently transfected for 48 h or 72 h using Lipofectamine 2000^TM^ (Invitrogen) according to manufacturer’s instructions with pMSCV-miR146 (or pMSCV-control plasmid) or with oligo-cel-miR-238 (containing the mature sequence of *C. elegans* miR-238). A pool of 4 siRNAs (SMARTpool from Dharmacon) was used to knockdown Ago2 expression. A custom-designed siRNA duplex directed against green fluorescent protein (siGFP) was used as negative control for siAgo2 and *cel-miR-238* experiments. Culture media were changed 8 h after transfection for complete DMEM media, in order to remove transfection reagents and avoid possible contamination of the incubation media with untransfected oligonucleotides.

### Microarray profiling

MIN6B1 cells were incubated for 48 h in full DMEM complemented with exosome-free FCS, in the absence or presence of cytokines. At the end of the incubation, the media were collected for exosome isolation and total RNA was extracted from both exosomes and cells. Global miRNA expression profiling was carried out at the Genomic Technologies Facility of the University of Lausanne using the Agilent Technologies miRNA Gene Microarrays. The microarrays included probes for 650 mouse miRNAs listed on http://www.mirbase.org/ (2010). For each condition, 100 ng of total cellular or exosomal RNA was analyzed. Computational analysis of optical signal-to-noise ratio was performed using the software Feature Extraction (version 10.5.1.1). A miRNA was considered “not detected” if the optical signal was too low or too variable. Quantile normalization with the software Genespring (version GX 11.0.2) was used to normalize all the samples. Comparisons between groups were made using Student *t* test, with adjusted *p*-values based on all the 650 miRNAs included in the microarray.

### Cell death

MIN6B1 or dissociated mouse islet cells were incubated for 2 min with 1 μg/ml of Hoechst 33342 (Invitrogen). Cells displaying pycnotic nuclei were scored under fluorescence microscopy (AxioCam MRc5, Zeiss). A minimum of five different fields per experiment for a total of at least thousand cells were counted for each experimental condition.

### Cell proliferation

MIN6B1 cells were seeded on poly-L-lysine-laminin-coated glass coverslips. BrdU (Roche) was added to the incubation medium for the last 6 h of culture. Thereafter, MIN6B1 cells were fixed in cold methanol and permeabilized using PBS supplemented with 0.5% saponin for 15 minutes. The coverslips were incubated in blocking buffer (PBS containing 0.5% saponin and 1% BSA) for 30 min and then sequentially exposed to a mouse anti-BrdU antibody (diluted 1/1400, Cell Signaling) for 1 h and to a goat anti-mouse Alexa Fluor 555 antibody (diluted 1/400, Invitrogen) for another 1 h. Finally, the cell nuclei were stained with Hoechst 33342 (1 μg/ml, Invitrogen) for 1 minute. Coverslips were mounted on microscope glass slides with Fluor-Save mounting medium (VWR International SA) and were visualized with a Zeiss Axiovision fluorescence microscope.

### Insulin secretion

MIN6B1 were pre-incubated at 2 mM glucose for 30 min in Krebs-Ringer bicarbonate buffer containing 25 mM HEPES (KRBH; pH 7.4) and 0.1% BSA. The cells were then incubated for 45 min in KRBH, 0.1% BSA at 2 or 20 mM glucose. At the end of the incubation, the media were collected for insulin measurement and the cells recovered in acidified ethanol (0.2 mM HCl in 75% ethanol) or in lysis buffer for the determination of cellular insulin or protein content, respectively. Insulin levels were measured by ELISA (Mercodia) and proteins by Bradford (BioRad).

### Statistical analysis

Data are expressed as means ± SD. Statistical significances were determined using one-way ANOVA of the means, followed by post-hoc Dunnett’s or Tukey’s test (GraphPad Software, USA). For the microarray analysis, comparisons between groups were made using Student *t* test, with adjusted *p*-values based on the Benjamini-Hochberg false discovery rate procedure taking in to account all the 650 miRNAs included in the microarray.

## Additional files

Additional file 1: Figure S1. Overexpression of miR-146a in MIN6B1 cells. MIN6B1 cells were transfected with a control pMSCV plasmid (Ctl) or with a plasmid coding for miR-146a (m146a). The level of miR-146a in cell extracts was measured by qPCR, 48 h after transfection. Results were normalized to U6 content and expressed as Fold change vs Ctl. *Significantly different from control condition p ≤ 0.05 by Student’s *t*-test. Additional file 2: Table S1. Microarray results. Exosomes were isolated from the culture media (complemented with exosome-free FCS) of MIN6B1 cells treated or not with cytokines for 48 h. Exosomal and cellular miRNA content of MIN6B1 cells were determined by microarray. Additional file 3: Figure S2. Confirmation of microarray results by qPCR. Exosomes were isolated from the culture media (complemented with exosome-free FCS) of MIN6B1 cells. A) The level of miR-32, miR-142-3p, miR-194 and miR-451 in exosomes and in cell extracts of MIN6B1 cells was measured by qPCR. B) The level of let-7e, miR-212-3p, miR-546 and miR-710 in exosomes from MIN6B1 treated or not with cytokines for 48 h cells was determined by qPCR. Results are expressed as Fold vs Cell or Ctl content. *Significantly different from control condition p ≤ 0.05 by Student’s *t*-test. Additional file 4: Figure S3. Exosomes of untreated MIN6B1 cells or media in which exosomes are resuspended do not affect recipient beta-cell survival. A) Exosomes were isolated from the media of untreated MIN6B1 cells cultured for 48 h. Recipient MIN6B1 cells were incubated without (NT) or with exosomes (Exo-ctl). B) MIN6B1 cells were incubated without (NT) or with medium fraction (MF) in which exosomes were resuspended. Cell death was assessed by scoring the cells displaying pycnotic nuclei upon Hoechst staining. Additional file 5: Figure S4. Efficiency of Ago2 silencing in MIN6B1 cells and in mouse islet cells. A) MIN6B1 or B) Dispersed mouse islet cells were transfected with siGFP or siAgo2. Cells were harvested 72 h after transfection. The level of Ago2 was measured by qRT-PCR and normalized to 18S. *Significantly different from control condition p ≤ 0.05 by Student’s *t*-test. Additional file 6: Figure S5. Ago2 inactivation in recipient beta-cells prevents apoptosis induced by exosomes of cytokine-treated cells. Exosomes were isolated from the culture media supplemented with exosome-free FCS of MIN6B1 cells treated for 0 h (Exo-Ctl), 24 h (Exo-cyt 24 h) or 48 h (Exo-cyt 48 h) with cytokines. Recipient MIN6B1 were transfected with siCtrl or siAgo2 and incubated for 72 h with the different exosome preparations. Apoptosis was assessed by scoring the cells displaying pycnotic nuclei upon Hoechst staining. *Significantly different from the respective control condition (siCtrl-Exo-Ctl), # Significantly different from the respective control condition (siCtrl-Exo-cyt24h or siCtrl-Exo-cyt48h). p ≤ 0.05 by ANOVA followed by Tukey’s post-hoc test.
