# Supplementary figures and images for: Horizontal transfer of exosomal microRNAs transduce apoptotic signals between pancreatic beta-cells
Source: Cell Commun Signal. 2015 Mar 19;13:17. doi: 10.1186/s12964-015-0097-7 (PMC4371845; doi:10.1186/s12964-015-0097-7)

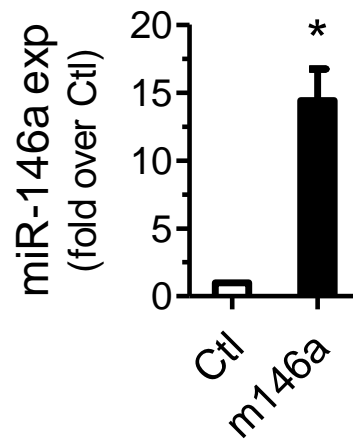

**Figure S1 : Overexpression of miR-146a in MIN6B1 cells**

Supplement: Additional file 1: Figure S1. — Overexpression of miR-146a in MIN6B1 cells. MIN6B1 cells were transfected with a control pMSCV plasmid (Ctl) or with a plasmid coding for miR-146a (m146a). The level of miR-146a in cell extracts was measured by qPCR, 48 h after transfection. Results were normalized to U6 content and expressed as Fold change vs Ctl. *Significantly different from control condition p ≤ 0.05 by Student’s t-test. [file 12964_2015_97_MOESM1_ESM.pdf]

A)

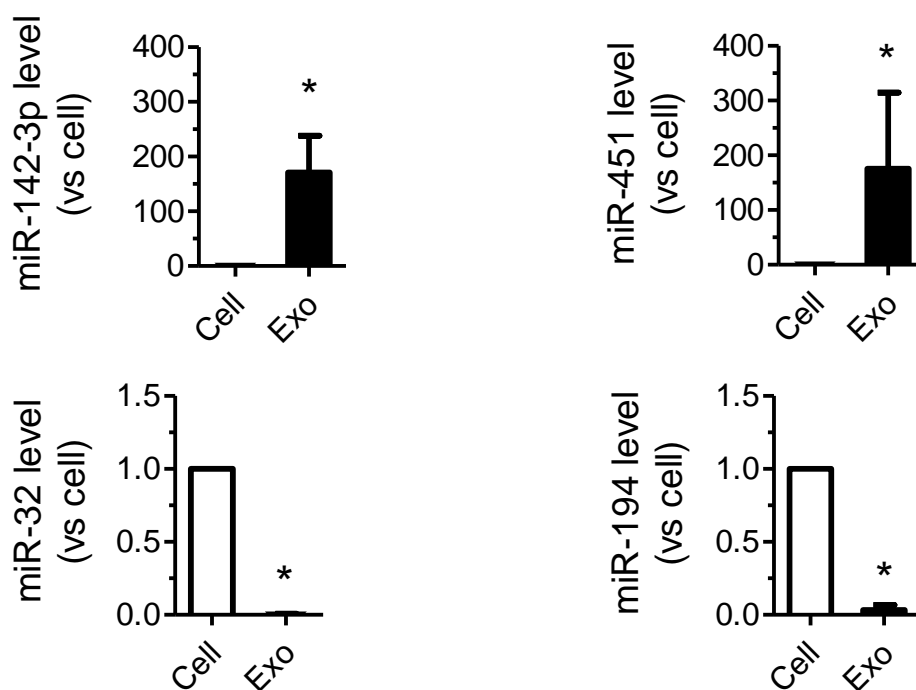

B)

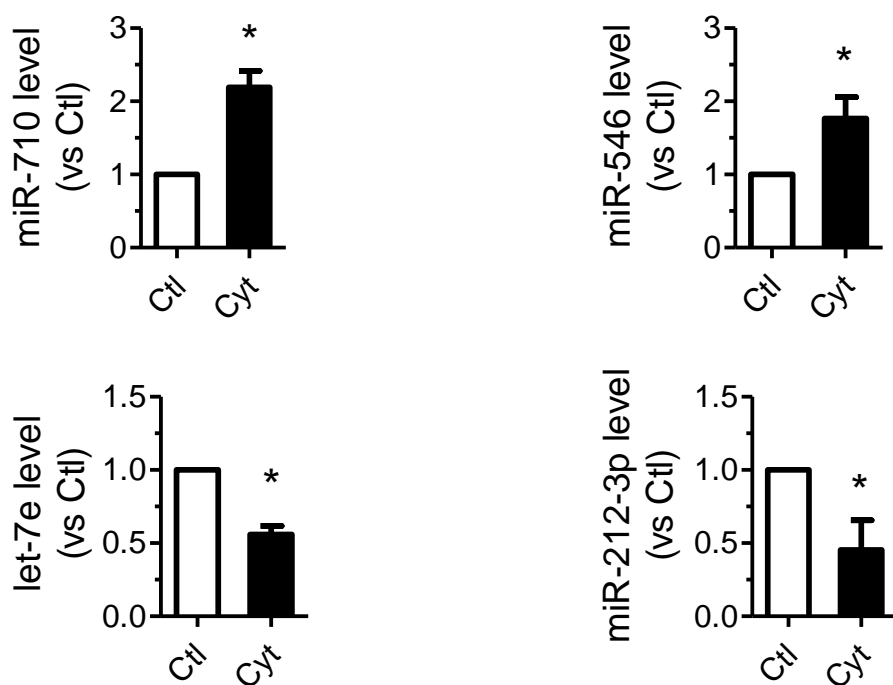

**Figure S2: Confirmation of microarray results by qPCR**

Supplement: Additional file 3: Figure S2. — Confirmation of microarray results by qPCR. Exosomes were isolated from the culture media (complemented with exosome-free FCS) of MIN6B1 cells. A) The level of miR-32, miR-142-3p, miR-194 and miR-451 in exosomes and in cell extracts of MIN6B1 cells was measured by qPCR. B) The level of let-7e, miR-212-3p, miR-546 and miR-710 in exosomes from MIN6B1 treated or not with cytokines for 48 h cells was determined by qPCR. Results are expressed as Fold vs Cell or Ctl content. *Significantly different from control condition p ≤ 0.05 by Student’s t-test. [file 12964_2015_97_MOESM3_ESM.pdf]

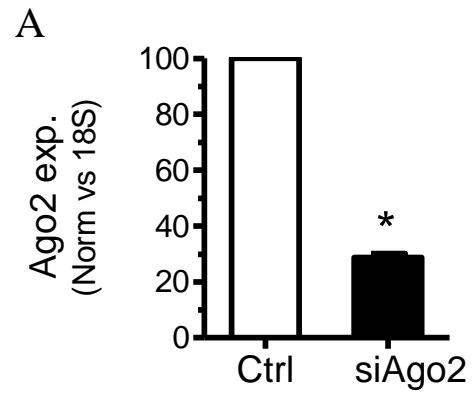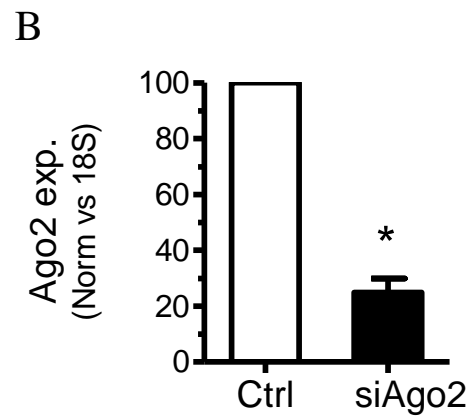

**Figure S4 : Efficiency of siRNA directed against Ago2**

Supplement: Additional file 5: Figure S4. — Efficiency of Ago2 silencing in MIN6B1 cells and in mouse islet cells. A) MIN6B1 or B) Dispersed mouse islet cells were transfected with siGFP or siAgo2. Cells were harvested 72 h after transfection. The level of Ago2 was measured by qRT-PCR and normalized to 18S. *Significantly different from control condition p ≤ 0.05 by Student’s t-test. [file 12964_2015_97_MOESM5_ESM.pdf]
