# Supplementary material for: Horizontal transfer of exosomal microRNAs transduce apoptotic signals between pancreatic beta-cells
Source: Cell Commun Signal. 2015 Mar 19;13:17. doi: 10.1186/s12964-015-0097-7 (PMC4371845; doi:10.1186/s12964-015-0097-7)
Supplement: Additional file 4: Figure S3. — Exosomes of untreated MIN6B1 cells or media in which exosomes are resuspended do not affect recipient beta-cell survival. A) Exosomes were isolated from the media of untreated MIN6B1 cells cultured for 48 h. Recipient MIN6B1 cells were incubated without (NT) or with exosomes (Exo-ctl). B) MIN6B1 cells were incubated without (NT) or with medium fraction (MF) in which exosomes were resuspended. Cell death was assessed by scoring the cells displaying pycnotic nuclei upon Hoechst staining. [file 12964_2015_97_MOESM4_ESM.pdf]

A

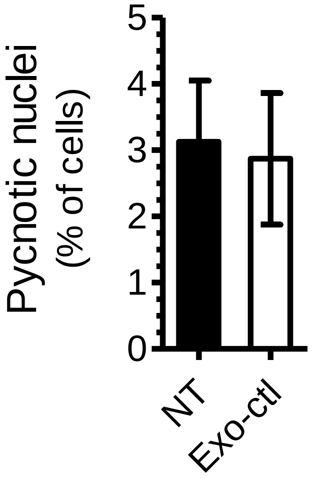

B

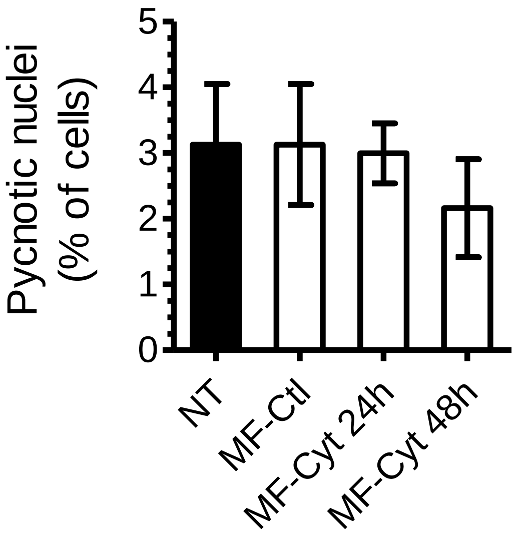

Figure S3: Exosomes of untreated MIN6B1 cells or media fraction in which exosomes were resuspended do not affect recipient naïve beta-cell survival
