# Supplementary material for: Horizontal transfer of exosomal microRNAs transduce apoptotic signals between pancreatic beta-cells
Source: Cell Commun Signal. 2015 Mar 19;13:17. doi: 10.1186/s12964-015-0097-7 (PMC4371845; doi:10.1186/s12964-015-0097-7)
Supplement: Additional file 6: Figure S5. — Ago2 inactivation in recipient beta-cells prevents apoptosis induced by exosomes of cytokine-treated cells. Exosomes were isolated from the culture media supplemented with exosome-free FCS of MIN6B1 cells treated for 0 h (Exo-Ctl), 24 h (Exo-cyt 24 h) or 48 h (Exo-cyt 48 h) with cytokines. Recipient MIN6B1 were transfected with siCtrl or siAgo2 and incubated for 72 h with the different exosome preparations. Apoptosis was assessed by scoring the cells displaying pycnotic nuclei upon Hoechst staining. *Significantly different from the respective control condition (siCtrl-Exo-Ctl), # Significantly different from the respective control condition (siCtrl-Exo-cyt24h or siCtrl-Exo-cyt48h). p ≤ 0.05 by ANOVA followed by Tukey’s post-hoc test. [file 12964_2015_97_MOESM6_ESM.pdf]

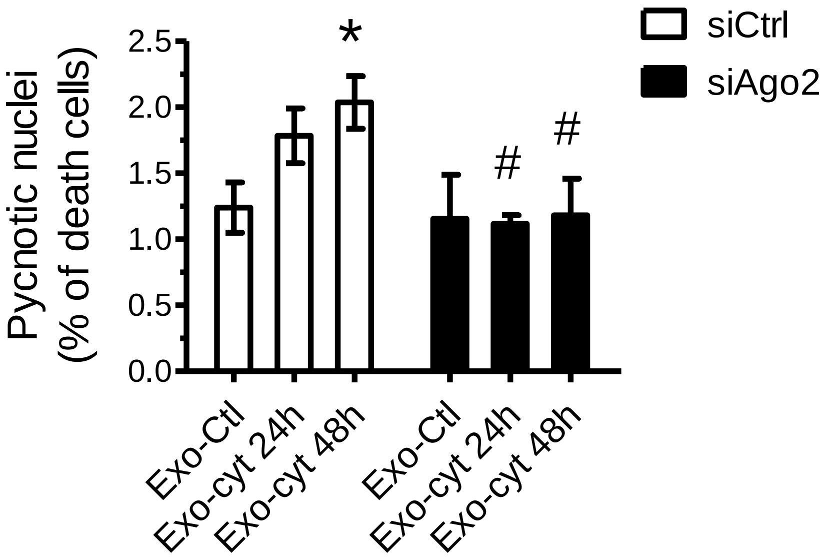

Figure S5 : Ago2 inactivation in recipient beta-cells prevents apoptosis induced by exosomes of cytokine-treated cells.
